# Supplementary material for: Synergistic Effect of Propidium Iodide and Small Molecule Antibiotics with the Antimicrobial Peptide Dendrimer G3KL against Gram-Negative Bacteria
Source: Molecules. 2020 Nov 30;25(23):5643. doi: 10.3390/molecules25235643 (PMC7730455; doi:10.3390/molecules25235643)
Supplement: Supplementary file 1 [file molecules-25-05643-s001.pdf]

Supporting information

Synergistic effect of propidium iodide and small molecule antibiotics with the antimicrobial peptide dendrimer G3KL against Gram-negative bacteria

Bee-Ha Gan[a)], Sacha Javor, [a)] Thilo Köhler[b)] and Jean-Louis Reymond [a)]*

[a)] Department of Chemistry and Biochemistry, University of Bern, Freiestrasse 3, 3012 Bern, Switzerland; [b)] Department of Microbiology and Molecular Medicine, University of Geneva, Service of Infectious Diseases, University Hospital of Geneva, Geneva, Switzerland

e-mail: jean-louis.reymond@dcb.unibe.ch

Table of Content

Supplemental Figures 2
References 17

Supplemental Figures

E. coli

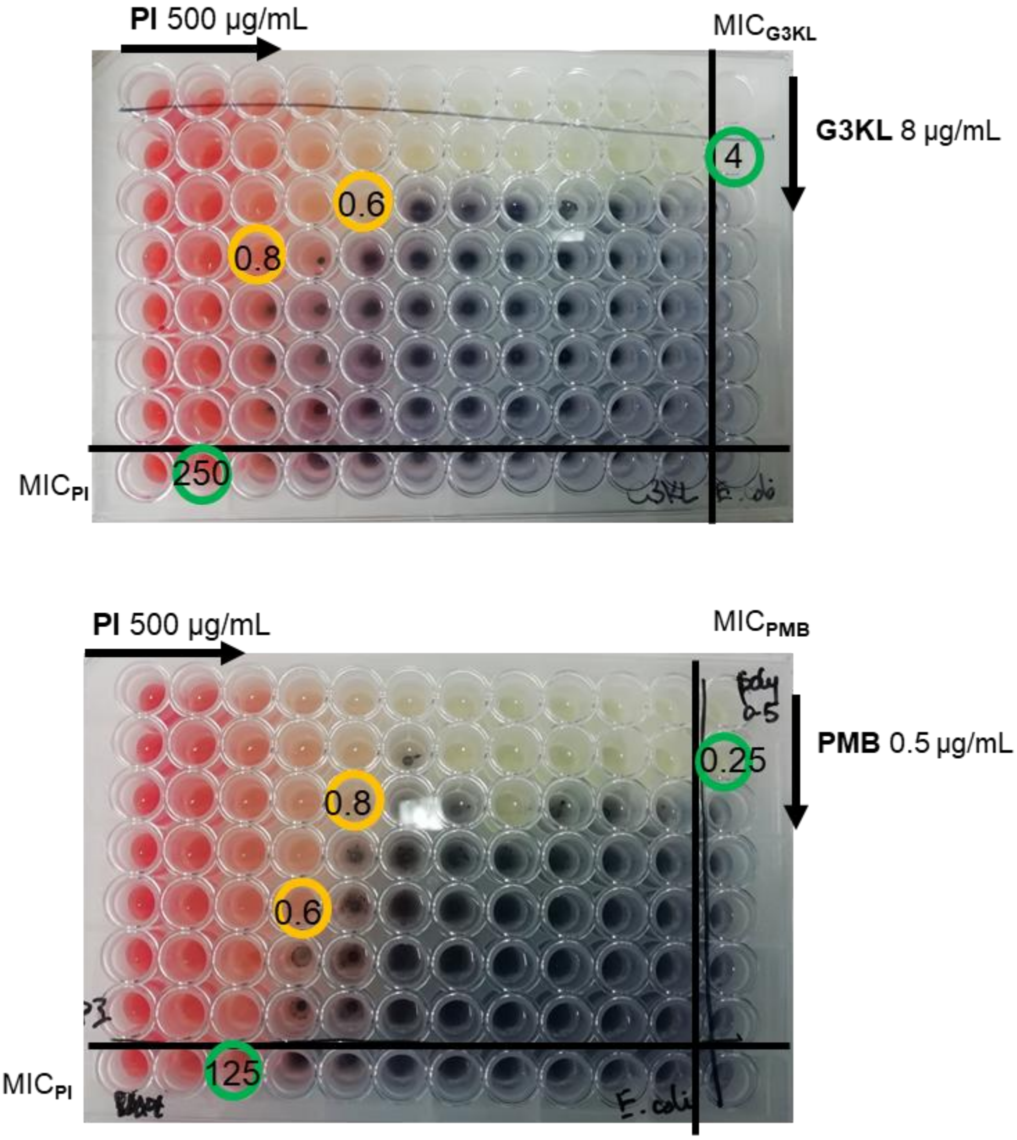

Figure S1. Checkerboard microtiter plate assay testing the combination of **G3KL**, PMB, and Cipro with **PI** in *E. coli*. Yellow circles: FIC_i of partial synergy and additive effects; Green circles: MIC values of the tested compounds.

A. baumannii

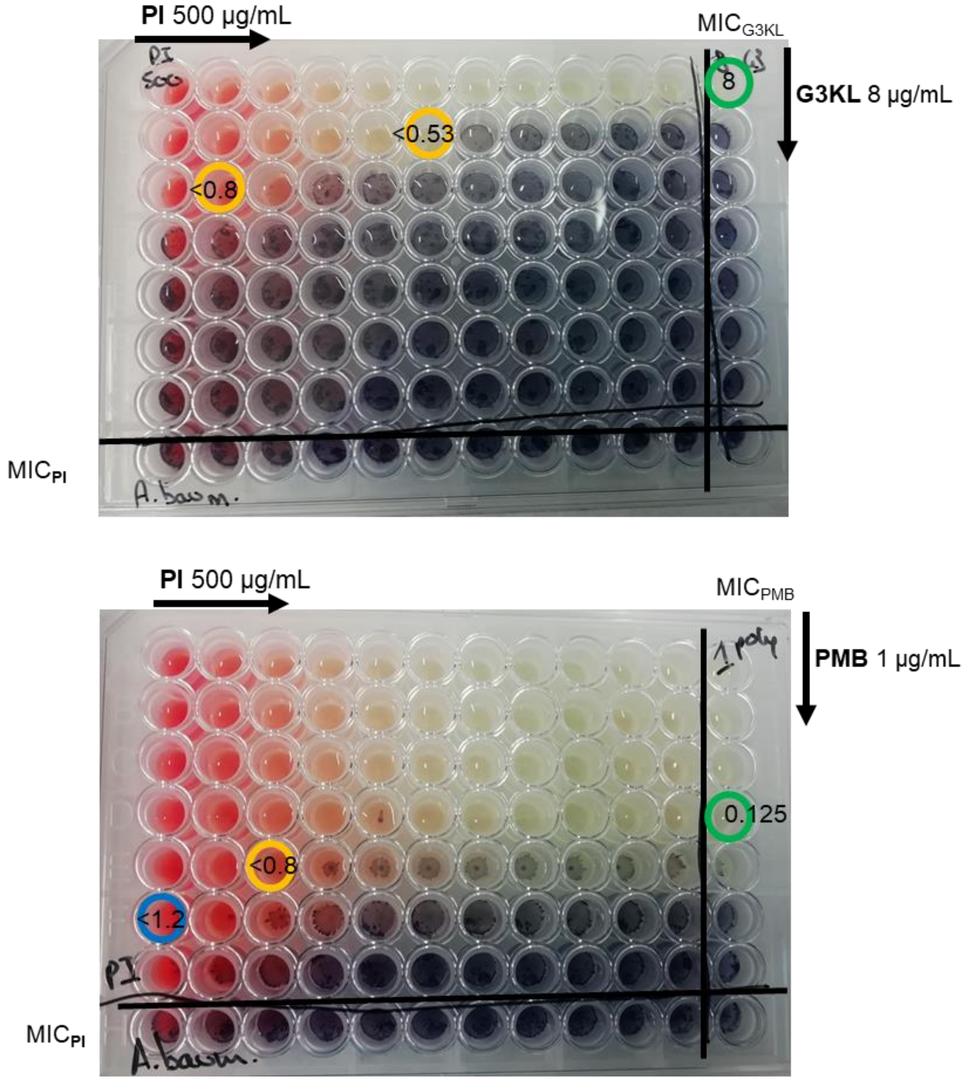

Figure S2. Checkerboard microtiter plate assay testing the combination of **G3KL**, PMB, and Cipro with **PI** in *A. baumannii*. Yellow circles: FIC_i of partial synergy; Blue circles: FIC_i of indifferent effect; Green circles: MIC values of the tested compounds.

K. pneumoniae

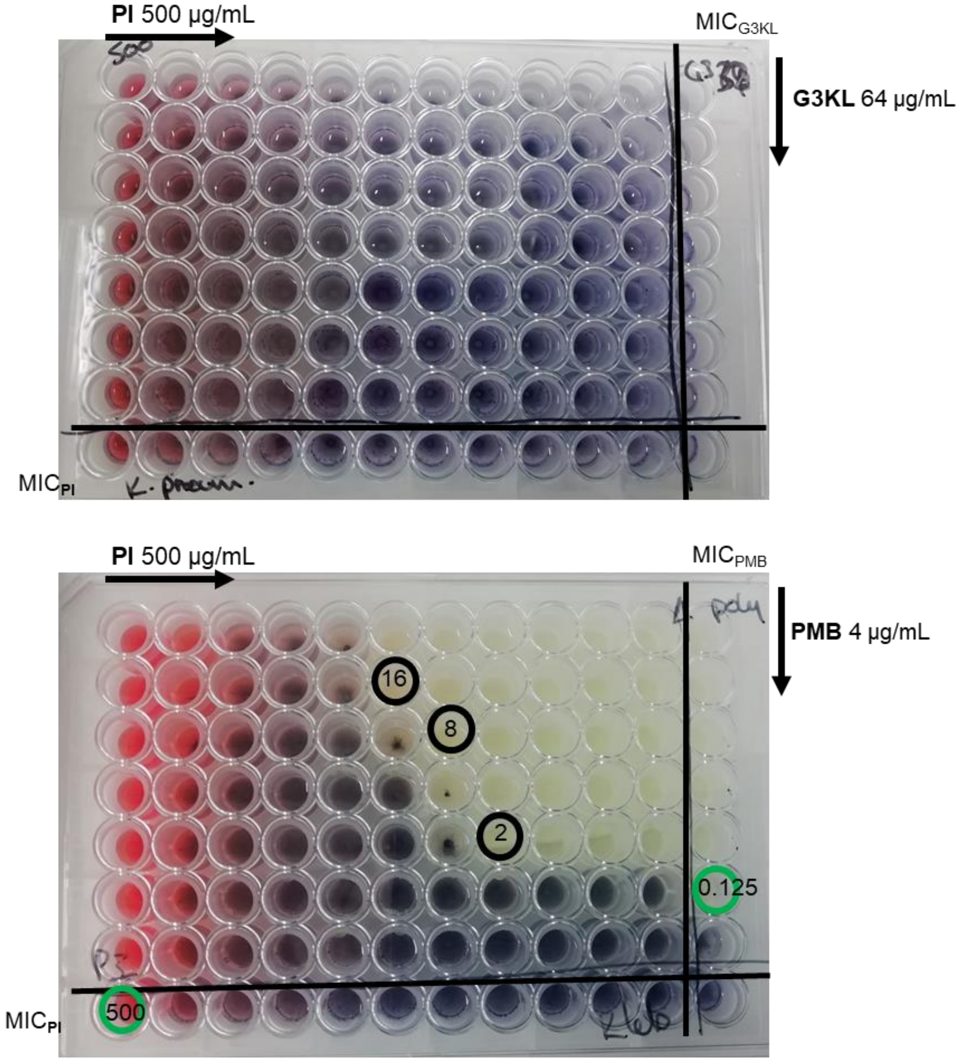

Figure S3. Checkerboard microtiter plate assay testing the combination of **G3KL**, PMB, and Cipro with **PI** in *K. pneumoniae*. Yellow circles: FIC$_i$ of additive effect; Black circles: FIC$_i$ of antagonist effect; Green circles: MIC values of the tested compounds.

MRSA

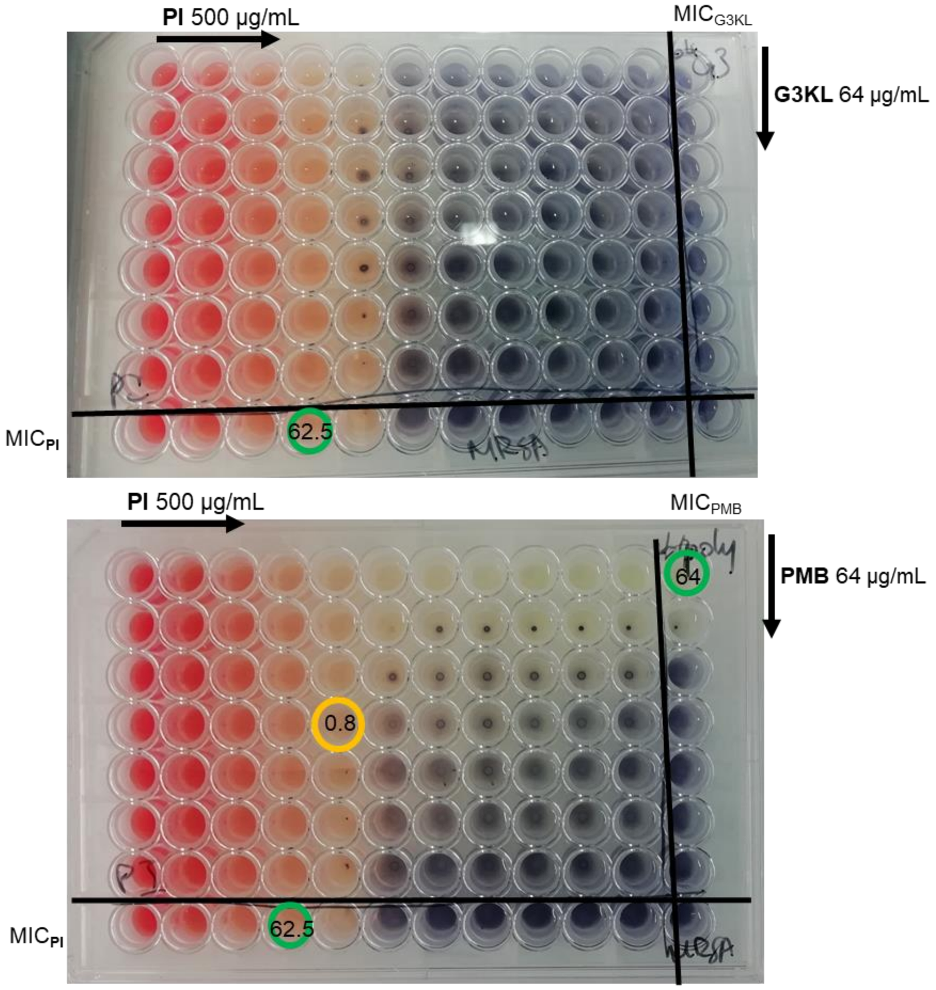

Figure S4. Checkerboard microtiter plate assay testing the combination of **G3KL**, PMB, and Cipro with **PI** in MRSA. Yellow circles: FIC_i of partial synergy; Green circles: MIC values of the tested compounds.

P. aeruginosa

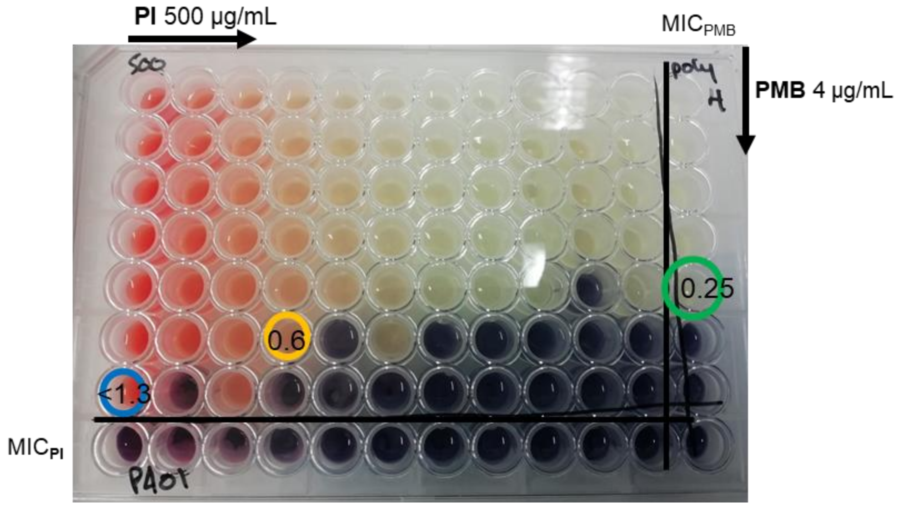

Figure S5. Checkerboard microtiter plate assay testing the combination of PMB and Cipro with **PI** in *P. aeruginosa*. Yellow circles: FIC_i of partial synergy; Blue circles: FIC_i of indifferent effect; Green circles: MIC values of the tested compounds.

P. aeruginosa

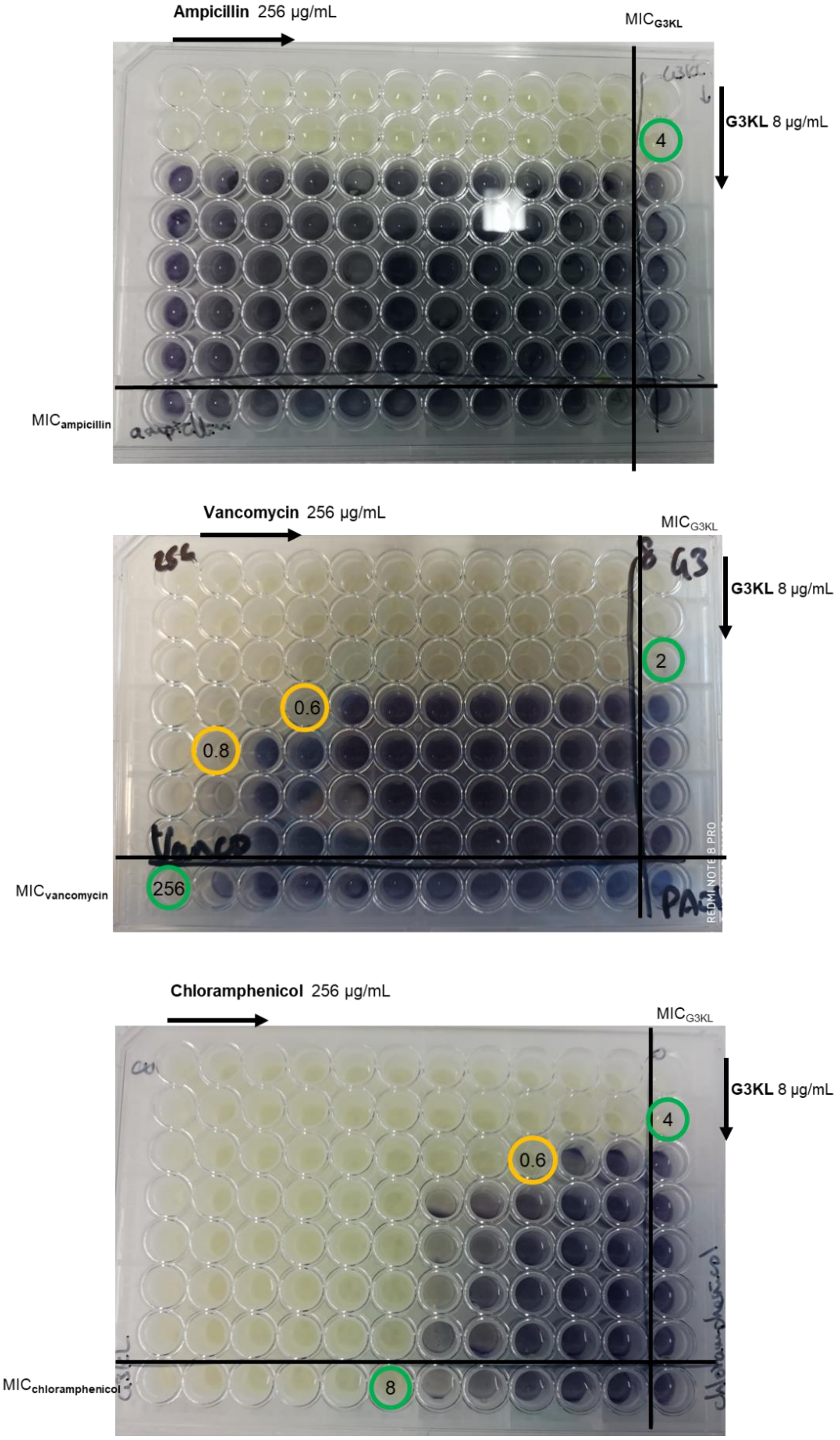

Figure S6. Checkerboard microtiter plate assay testing the combination of ampicillin, vancomycin, and chloramphenicol with **G3KL** in *P. aeruginosa*. Yellow circles: FIC_i of partial synergy; Green circles: MIC values of the tested compounds.

P. aeruginosa

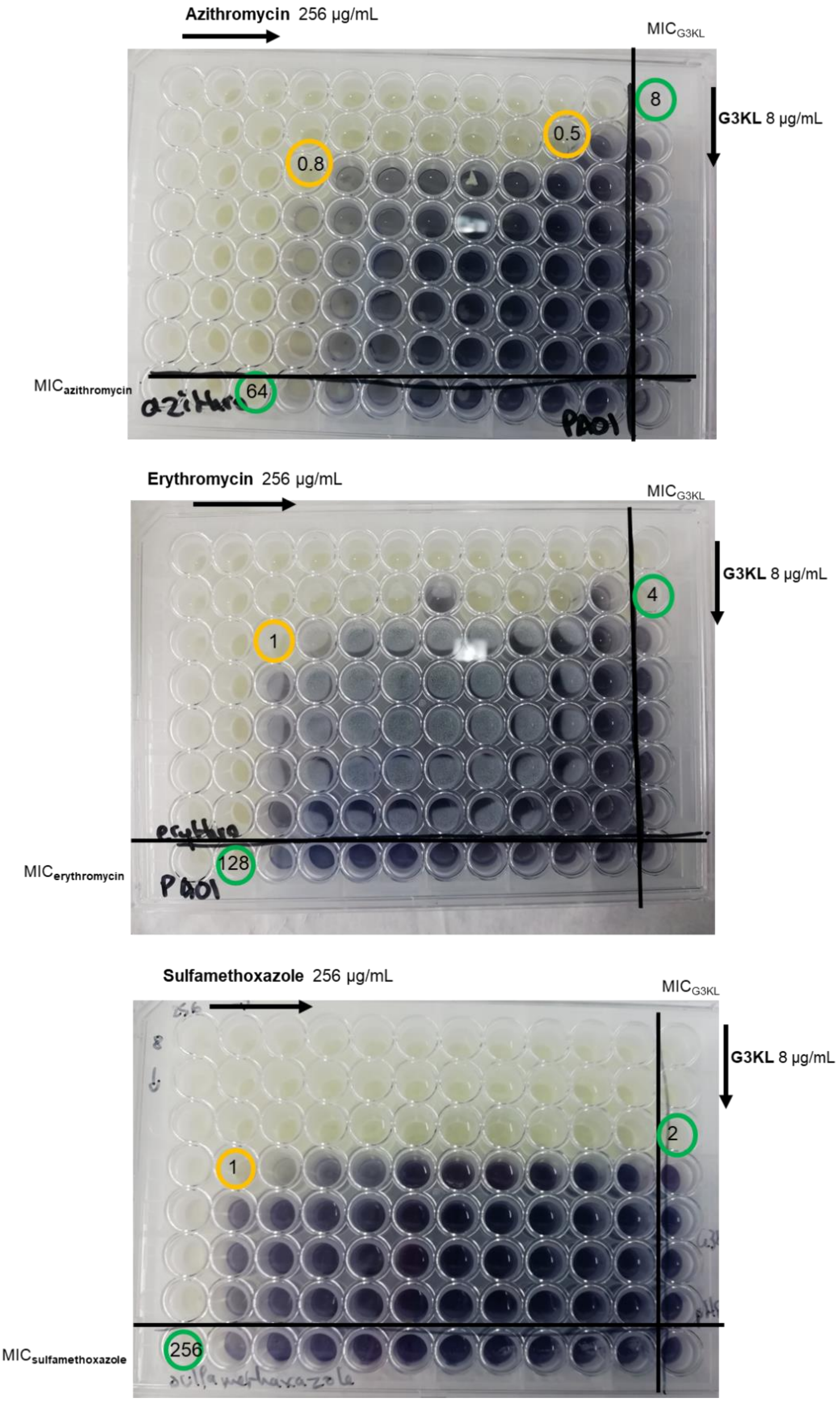

Figure S7. Checkerboard microtiter plate assay testing the combination of azithromycin, erythromycin, and sulfamethoxazole with **G3KL** in *P. aeruginosa*. Yellow circles: FIC_i of partial synergy; Green circles: MIC values of the tested compounds.

P. aeruginosa

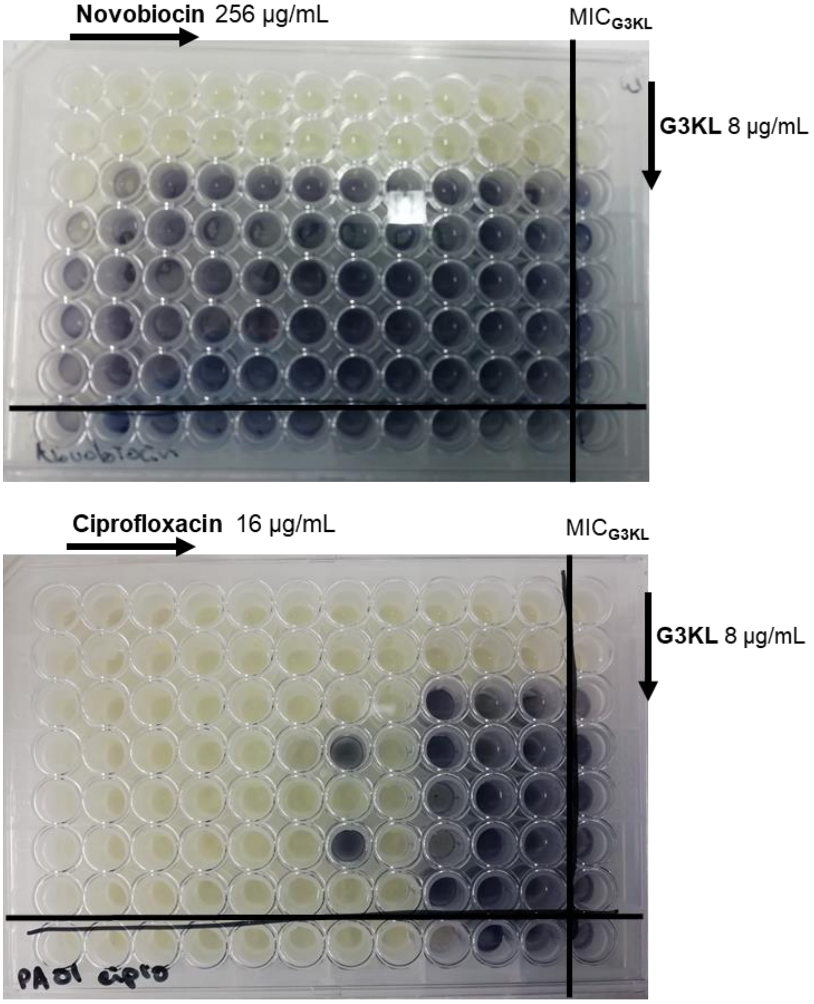

Figure S8. Checkerboard microtiter plate assay testing the combination of novobiocin, and ciprofloxacin with **G3KL** in *P. aeruginosa*.

P. aeruginosa

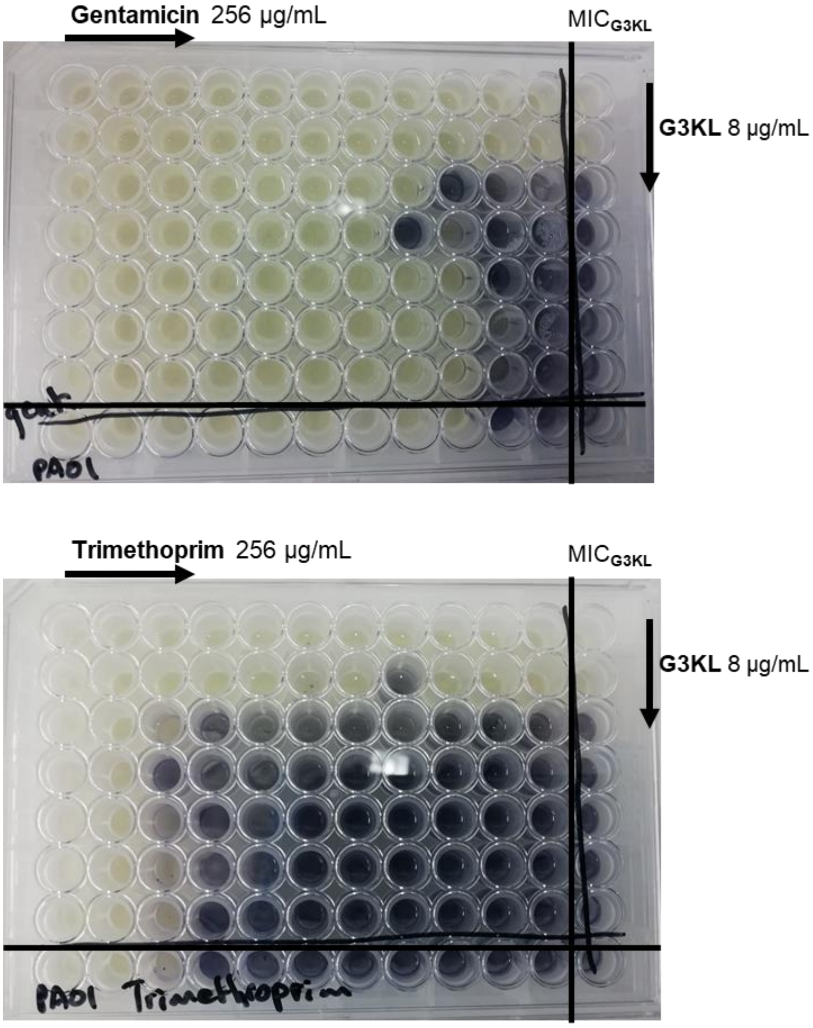

Figure S9. Checkerboard microtiter plate assay testing the combination of gentamicin and trimethoprim with **G3KL** in *P. aeruginosa*.

K. pneumoniae

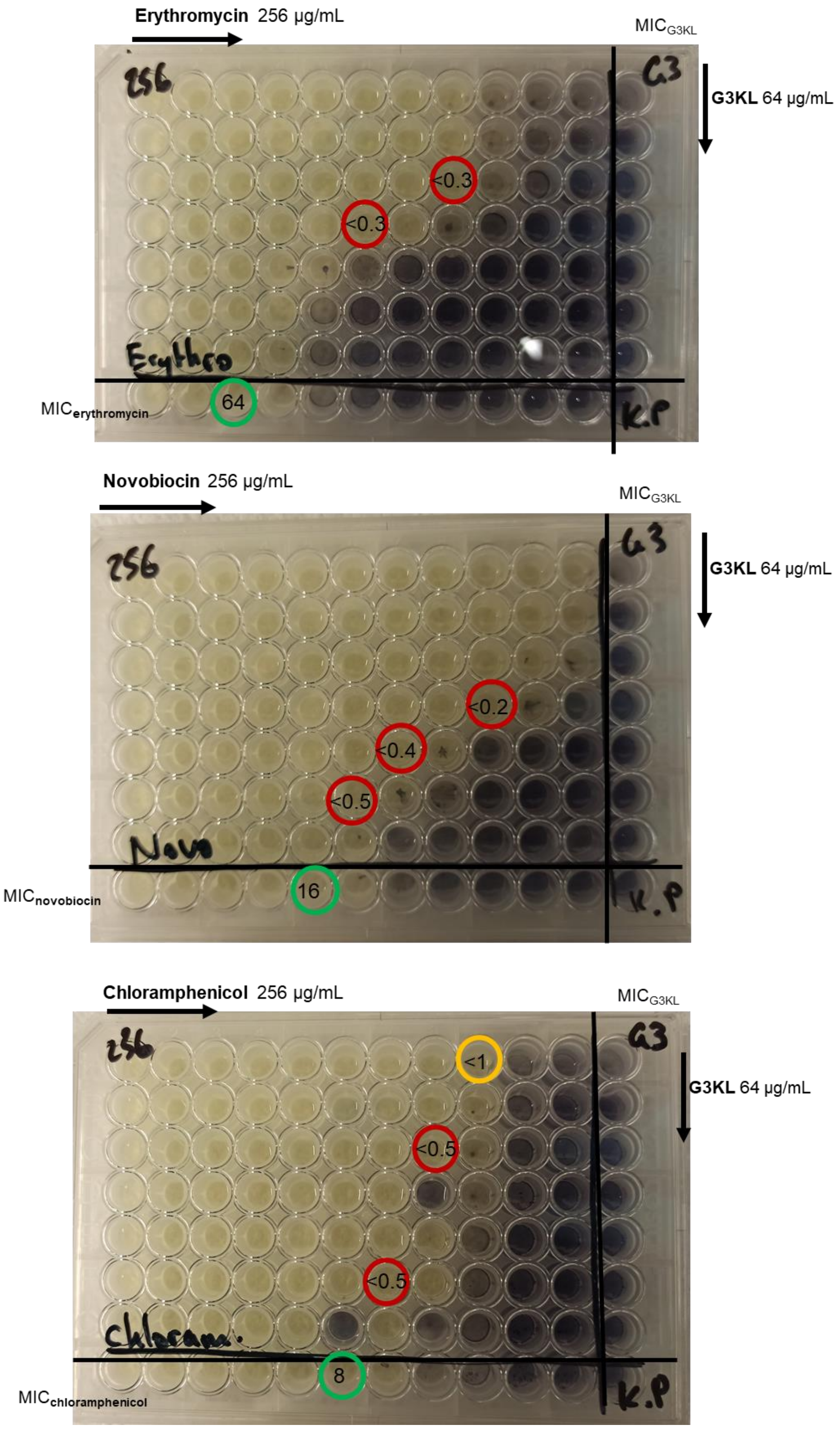

Figure S10. Checkerboard microtiter plate assay testing the combination of erythromycin, novobiocin, and chloramphenicol with **G3KL** in *K. pneumoniae*. Red circles: FIC_i of synergistic effect; Yellow: FIC_i of partial synergy; Green circles: MIC values of the tested compounds.

K. pneumoniae

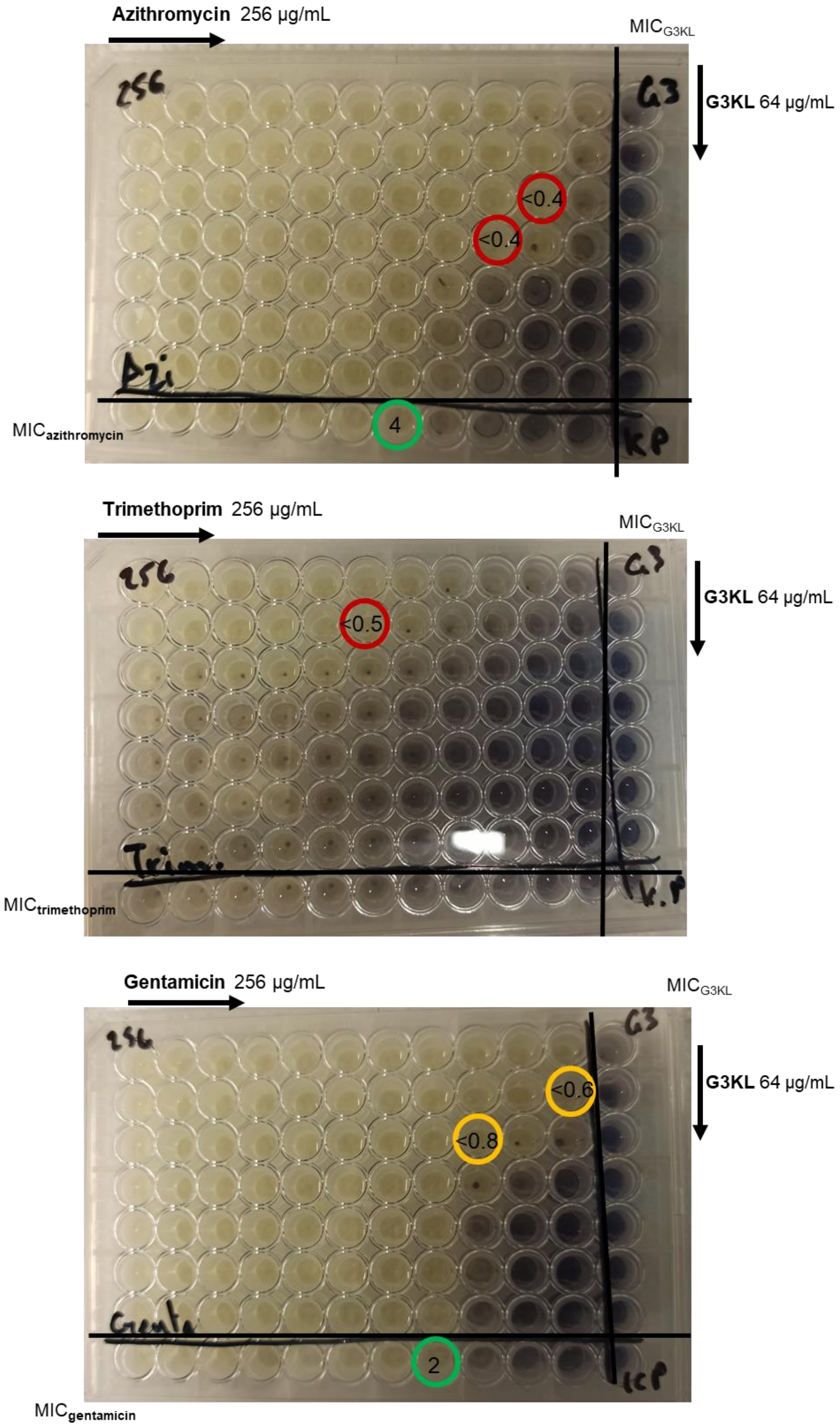

Figure S11. Checkerboard microtiter plate assay testing the combination of azithromycin, trimethoprim, and gentamicin with **G3KL** against *K. pneumoniae*. Red circles: FIC_i of synergistic effect; Yellow circles: FIC_i of partial synergy and additive effects; Green circles: MIC values of the tested compounds.

K. pneumoniae

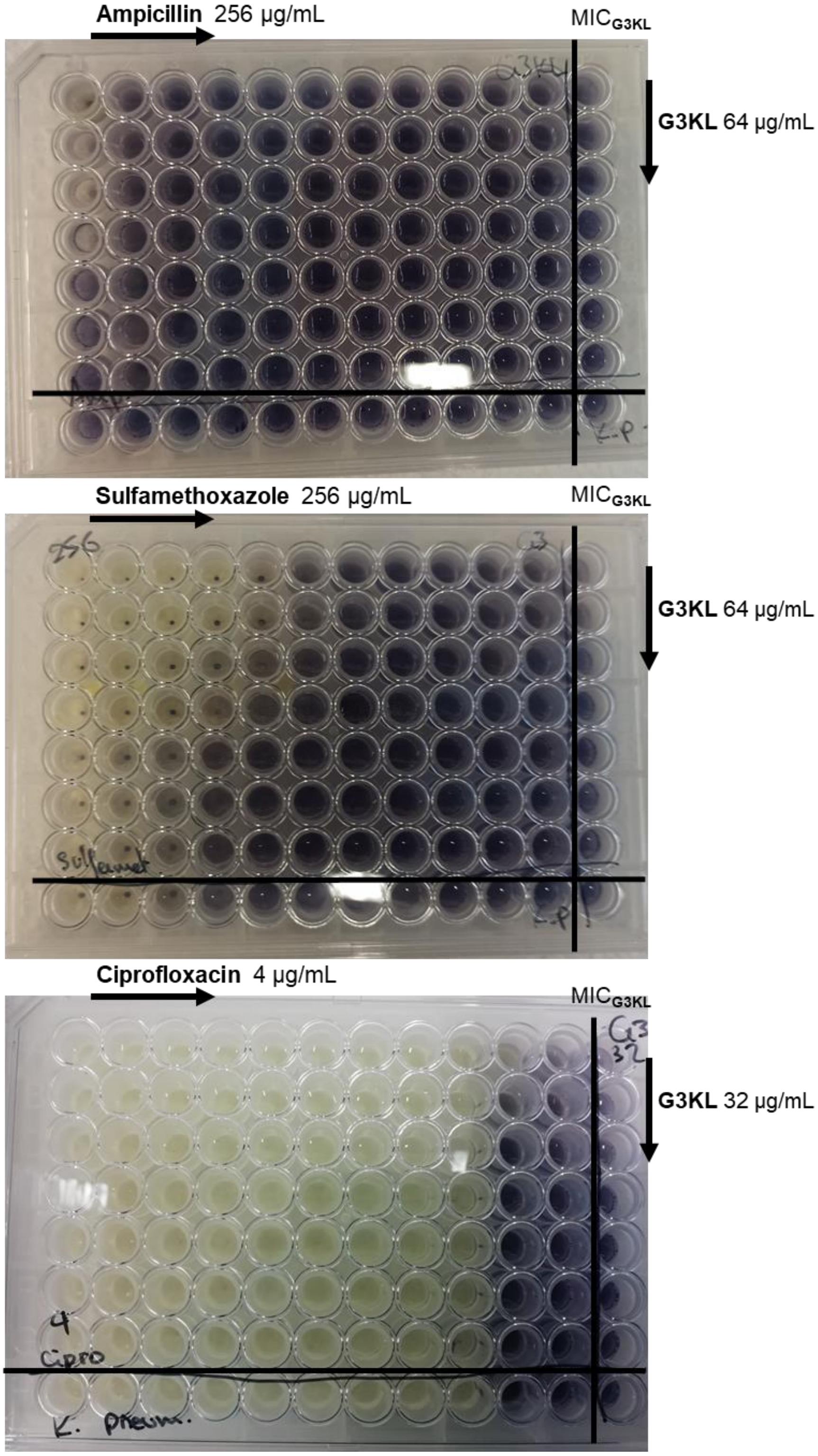

Figure S12. Checkerboard microtiter plate assay testing the combination of ampicillin, sulfamethoxazole, and ciprofloxacin with **G3KL** in *K. pneumoniae*.

MRSA

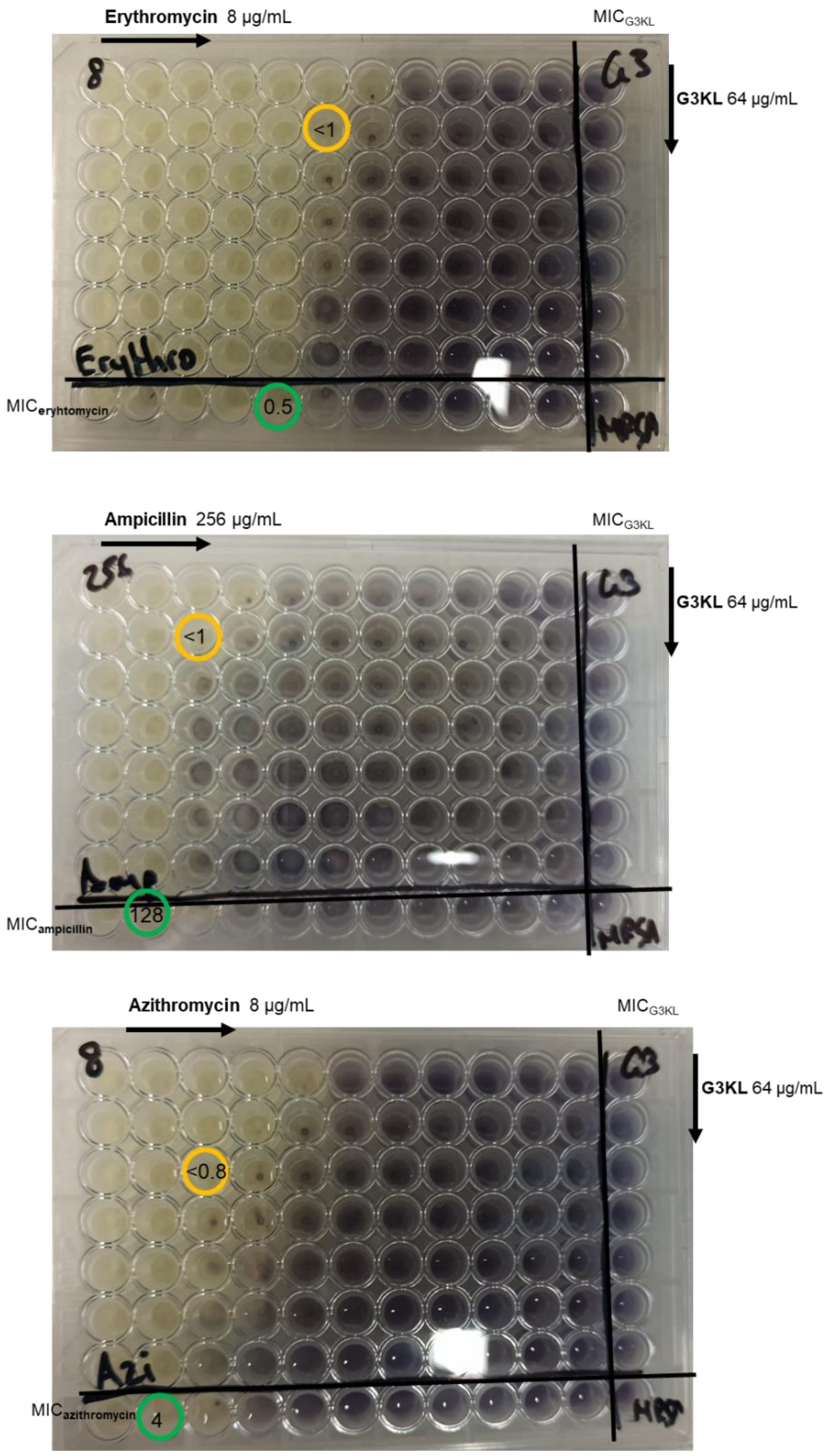

Figure S13. Checkerboard microtiter plate assay testing the combination of erythromycin, ampicillin, and azithromycin with **G3KL** in MRSA. Yellow circles: FIC_i of partial synergy and additive effects; Green circles: MIC values of the tested compounds.

MRSA

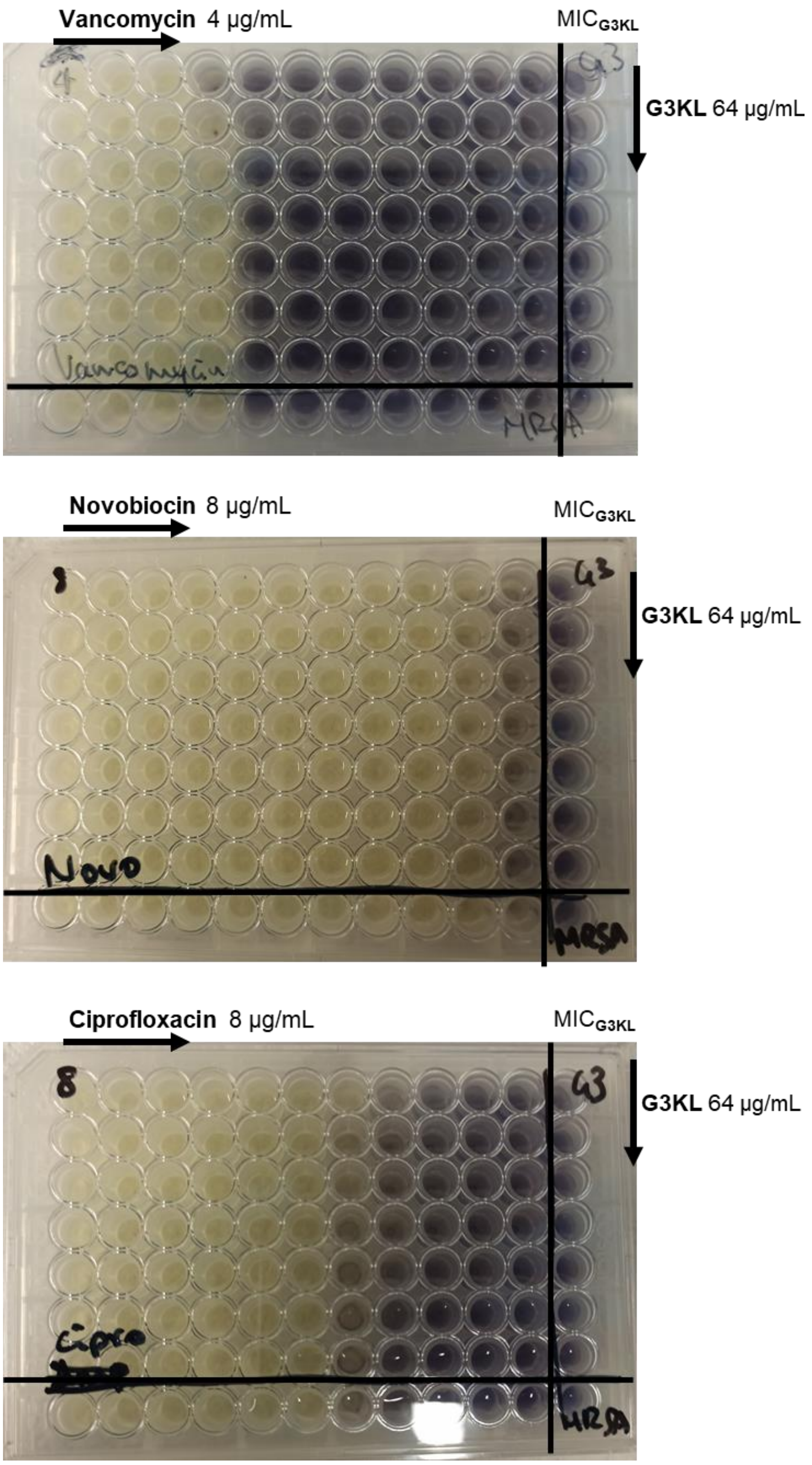

Figure S14. Checkerboard microtiter plate assay testing the combination of vancomycin, novobiocin, and ciprofloxcin with **G3KL** in MRSA.

MRSA

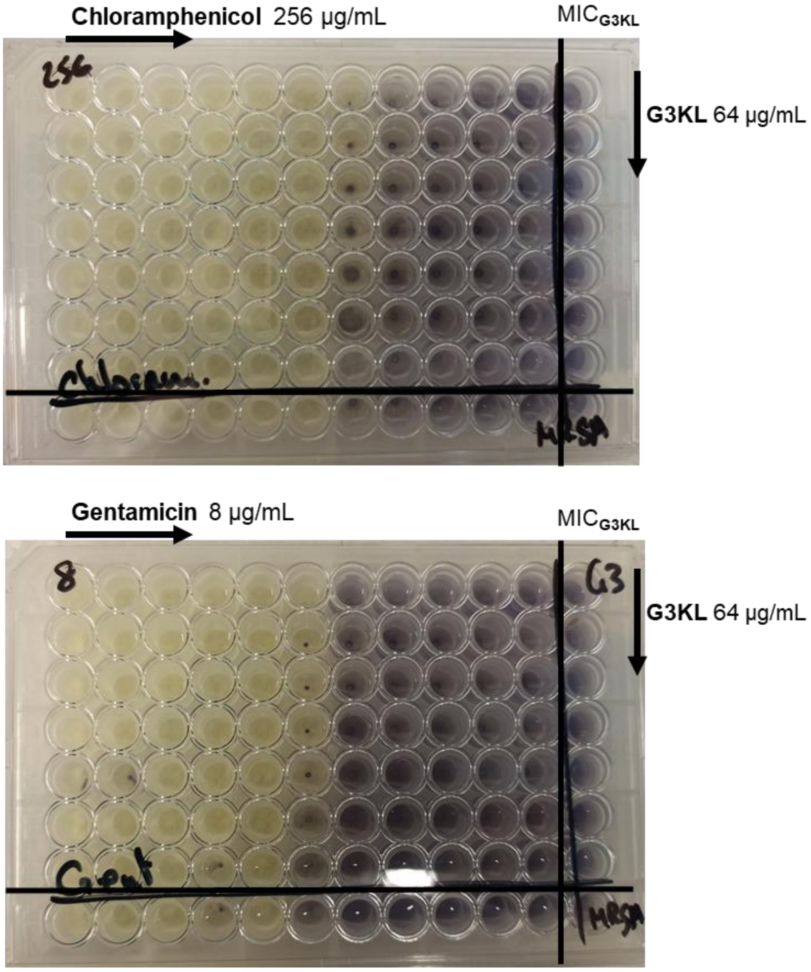

Figure S15. Checkerboard microtiter plate assay testing the combination of chloramphenicol and gentamicin with **G3KL** in MRSA.

MRSA

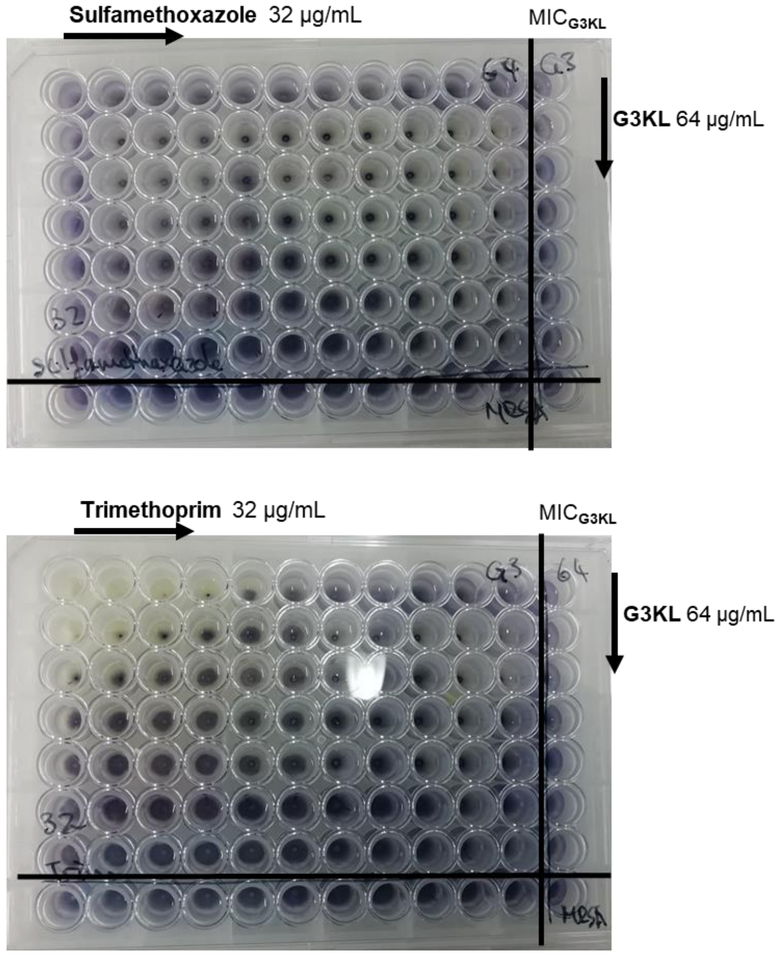

Figure S16. Checkerboard microtiter plate assay testing the combination of sulfamethoxazole and trimethoprim with **G3KL** in MRSA.

References

(1) Gan, B.-H.; Siriwardena, T. N.; Javor, S.; Darbre, T.; Reymond, J.-L. Fluorescence Imaging of Bacterial Killing by Antimicrobial Peptide Dendrimer G3KL. *ACS Infect. Dis.* **2019**, *5*, 2164–2173.

(2) Gopal, R.; Kim, Y. G.; Lee, J. H.; Lee, S. K.; Chae, J. D.; Son, B. K.; Seo, C. H.; Park, Y. Synergistic Effects and Antibiofilm Properties of Chimeric Peptides against Multidrug-Resistant Acinetobacter Baumannii Strains. *Antimicrob. Agents Chemother.* **2014**, *58*, 1622–1629.
